# Targeting endoplasmic reticulum stress: a novel therapeutic strategy for neuropathic pain

**DOI:** 10.3389/fpain.2026.1761647

**Published:** 2026-06-04

**Authors:** Yang Niu, Jiayu Han, Tao Song, Xueshu Tao

**Affiliations:** Department of Pain Medicine, The First Hospital of China Medical University, Shenyang, Liaoning, China

**Keywords:** endoplasmic reticulum stress, neuroinflammation, neuropathic pain, phytochemicals, unfolded protein response

## Abstract

Neuropathic pain (NP) is a chronic and debilitating condition arising from lesions or diseases of the somatosensory system. Increasing evidence identifies endoplasmic reticulum (ER) stress as a central mechanism contributing to NP pathogenesis. Activation of the unfolded protein response disrupts cellular homeostasis and promotes many pathological processes, including neuroinflammation, oxidative stress, apoptosis, and ferroptosis. Notably, ER stress signaling exhibits strong cell-type specificity, affecting neurons, glial cells, and immune cells across both peripheral and central nervous systems. Targeting ER stress has therefore emerged as a promising therapeutic strategy. However, ER stress signaling remains complex, and clinical translation is still limited. This review summarizes current mechanisms and highlights emerging ER stress-targeted therapies for NP.

## 1 Introduction

Neuropathic pain (NP) arises from injury or dysfunction of the somatosensory system and is characterized by spontaneous pain, hyperalgesia, and allodynia. Common etiologies include diabetic peripheral neuropathy, spinal cord injury, and postherpetic neuralgia (1). Epidemiological studies estimate that NP affects approximately 7%–10% of the global population, imposing a substantial burden on quality of life and healthcare systems (2). Despite the availability of first-line treatments such as gabapentinoids and antidepressants, clinical efficacy remains limited and is often accompanied by adverse effects (1, 3). These limitations highlight the urgent need to identify novel mechanisms and therapeutic targets.

The endoplasmic reticulum (ER) plays a fundamental role in protein folding, calcium regulation, and cellular homeostasis. Under pathological conditions, including oxidative stress and metabolic disturbance, misfolded proteins accumulate within the ER lumen, leading to ER stress (4–6). In response, cells activate the unfolded protein response (UPR), a signaling network mediated by three core sensors: PERK, IRE1*α*, and ATF6. While the UPR initially functions as a protective mechanism, prolonged or excessive activation can trigger inflammatory signaling, neuronal dysfunction, and cell death (5, 6).

Recent studies increasingly suggest that ER stress is closely involved in the development and maintenance of NP. Dysregulation of UPR signaling has been observed in both peripheral and central nervous systems following nerve injury. Importantly, ER stress interacts with multiple pathological processes, including neuroinflammation, synaptic plasticity, and oxidative stress (4), thereby amplifying pain signaling. In this context, targeting ER stress represents a promising strategy for improving NP management. This review aims to summarize the molecular and cellular mechanisms linking ER stress to NP and to discuss emerging therapeutic approaches that modulate ER stress signaling.

## 2 Endoplasmic reticulum stress and the unfolded protein response

The ER plays a central role in maintaining cellular proteostasis. When ER homeostasis is disrupted, misfolded proteins accumulate and activate ER stress signaling pathways (7, 8). In response, the UPR is initiated to restore ER function. Three major transmembrane sensors regulate the UPR:

### 2.1 PERK pathway

PERK is activated through dissociation from the chaperone protein GRP78. Activated PERK phosphorylates eukaryotic initiation factor 2*α* (eIF2*α*), leading to global suppression of protein translation while selectively enhancing translation of activating transcription factor 4 (ATF4). ATF4 subsequently induces expression of genes involved in stress adaptation, redox balance, and apoptosis (9, 10).

### 2.2 IRE1 pathway

IRE1 is an ER transmembrane kinase and endoribonuclease that activates the transcription factor X-box binding protein 1 (XBP1). Spliced XBP1 promotes transcription of genes involved in protein folding, ER-associated degradation, and lipid biosynthesis. In addition, IRE1 signaling can activate inflammatory pathways, including JNK and NF-*κ*B (6, 11).

### 2.3 ATF6 pathway

Upon ER stress, ATF6 translocates from the ER to the Golgi apparatus where it undergoes proteolytic cleavage. The active ATF6 fragment then enters the nucleus and promotes transcription of genes encoding ER chaperones and protein-folding enzymes (6). The UPR functions as a double-edged regulatory system. Under moderate or transient stress, the UPR restores proteostasis and supports cell survival. It helps cells adapt to injury. In contrast, sustained activation shifts signaling toward inflammation and cell death (6). This transition is highly relevant in NP. Early ER stress responses in neurons or glial cells may be protective. Chronic activation, however, promotes neuroinflammation and pain hypersensitivity.

## 3 Cell-Specific roles of ER stress in neuropathic pain

**Figure 1:**
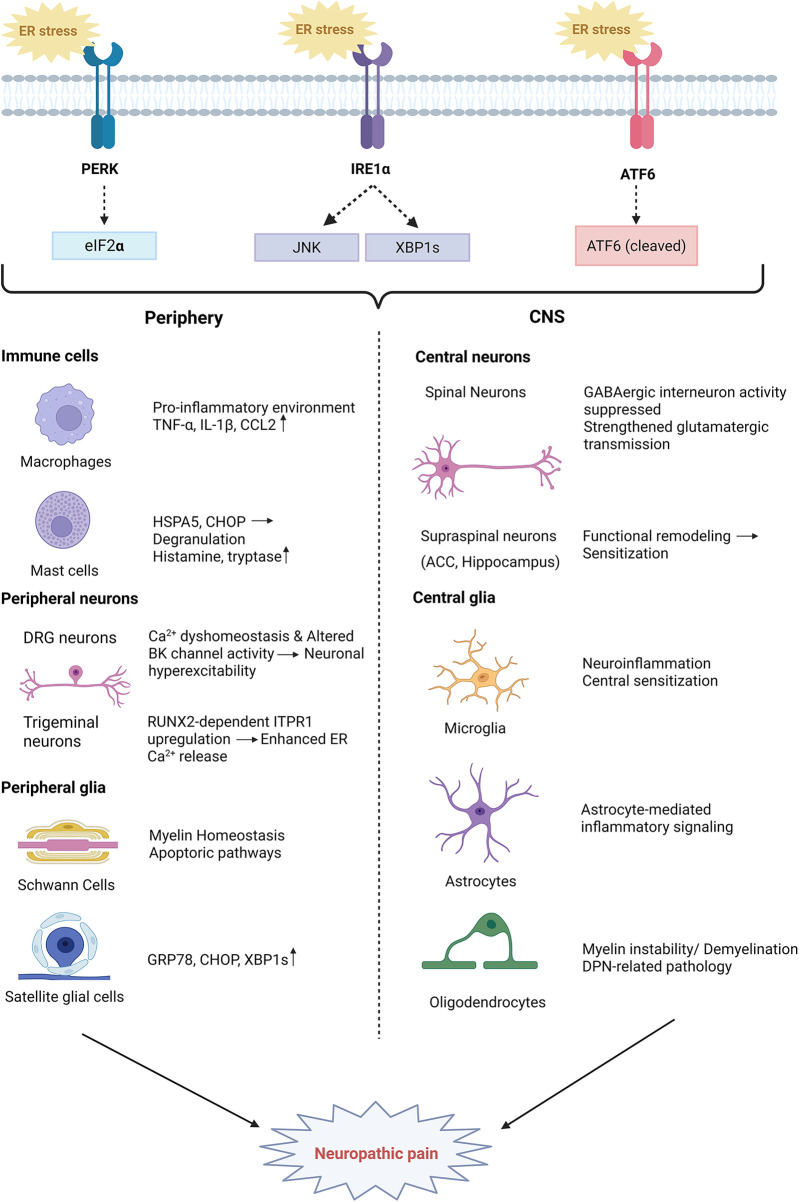
Overview of the cell-specific roles of endoplasmic reticulum stress in neuropathic pain. ER stress affects multiple cell types in both the peripheral and central nervous systems (CNS), including immune cells, peripheral sensory neurons, Schwann cells, satellite glial cells, central neurons, microglia, astrocytes, and oligodendrocytes. These responses lead to several major pathological consequences, such as enhanced inflammation, increased neuronal excitability, disrupted synaptic transmission, impaired myelin homeostasis, and glial reactivity. Peripheral changes promote the initiation and amplification of nociceptive signaling, whereas central changes further drive central sensitization and pain persistence. Together, these cell-specific and region-specific alterations contribute to the development and maintenance of neuropathic pain. Created in BioRender. Xueshu, T. (2026) https://BioRender.com/lmt89t7.

Although many studies have shown a close association between ER stress and NP, the underlying mechanisms remain complex. ER stress contributes to NP through several pathological processes, including neuroinflammation, autophagy, ferroptosis, oxidative stress, and apoptosis (12–15). However, most studies focus mainly on molecular pathways, while the cellular targets of ER stress in NP are less well understood. Therefore, this review summarizes current evidence from a cell-specific perspective to clarify how ER stress contributes to the development and maintenance of NP and to highlight potential cell-targeted therapeutic strategies (Figure 1).

### 3.1 Immune cells

Peripheral nerve injury rapidly activates immune cells and recruits them to the lesion site and dorsal root ganglia. These cells release inflammatory mediators such as TNF-α, IL-1β, and CCL2, which lower nociceptor activation thresholds and promote ectopic neuronal discharges (16). ER stress plays an important role in this immune response. In chemotherapy-induced neuropathic pain, paclitaxel activates the ER stress sensor IRE1*α* in macrophages, which promotes a pro-inflammatory microenvironment and contributes to pain development. Inhibition or genetic deletion of IRE1*α* reduces dorsal root ganglion neuroinflammation and alleviates pain behaviors in mice (17). In diabetic peripheral neuropathy, persistent metabolic stress induces ER stress and mitochondrial dysfunction in mast cells, leading to degranulation and release of inflammatory mediators such as histamine and tryptase, which disrupt the neural microenvironment (18). Together, these findings indicate that ER stress in immune cells acts as an important link between inflammation and neuronal sensitization, thereby contributing to the initiation and persistence of neuropathic pain.

### 3.2 Peripheral Glia: Schwann cells and satellite glial cells

#### 3.2.1 Schwann cells

Schwann cells (SCs) maintain peripheral nerve integrity and support axonal function. Pathological conditions such as diabetes and chemotherapy disrupt ER proteostasis and activate the UPR. This process alters myelin homeostasis, promotes inflammatory signaling, and leads to neuronal dysfunction (19). In diabetic peripheral neuropathy, hyperglycemia induces ER stress in SCs and activates apoptotic pathways that damage peripheral nerves (20). In addition, the chemotherapeutic drug bortezomib induces ER stress in SCs, disrupts myelin gene expression, and promotes macrophage recruitment, thereby contributing to chemotherapy-induced neuropathy (21). However, deletion of the ER stress sensor PERK in SCs does not prevent diabetic neuropathy, whereas PERK signaling is required for the therapeutic action of cemdomespib through activation of Nrf2 signaling (22). Overall, ER stress in SCs is closely associated with peripheral nerve dysfunction and may serve as a promising therapeutic target.

#### 3.2.2 Satellite glial cells

Satellite glial cells (SGCs) in the dorsal root ganglia (DRG) regulate neuronal excitability and play an important role in peripheral sensitization during NP (16). In a spinal nerve ligation model, ER stress markers such as GRP78, CHOP, and spliced XBP1 are significantly increased in the injured DRG. Immunohistochemical analysis shows that these markers are expressed in both DRG neurons and SGCs. Pharmacological inhibition of ER stress with salubrinal reduces CHOP expression and alleviates pain hypersensitivity. In contrast, local administration of the ER stress inducer tunicamycin in the DRG rapidly induces pain hypersensitivity (23). These findings suggest that ER stress within the DRG microenvironment, involving neurons and SGCs, contributes to NP development. However, direct mechanistic evidence linking ER stress specifically in SGCs to NP remains limited and requires further investigation.

### 3.3 Central glial cells: microglia, astrocytes, and oligodendrocytes

#### 3.3.1 Microglia

Microglia serve as the primary immune cells of the central nervous system and are critically involved in the development of neuropathic pain. Increasing evidence shows that ER stress in microglia promotes neuroinflammation and central sensitization. In central post-stroke pain models, ER stress in the thalamus activates UPR-dependent MAPK signaling and triggers reactive microglial responses (15). In sickle cell disease, elevated cell-free heme activates TLR4 signaling in spinal microglia, which induces oxidative stress and ER stress and drives hyperalgesia (24). Chemotherapeutic agents such as oxaliplatin can also induce ER stress in microglia and promote inflammatory responses (25). Importantly, several interventions alleviate pain by targeting microglial ER stress. For example, kinsenoside suppresses ER stress through the IL-10/p-STAT3/SOCS3 pathway (26), while antioxidant metals such as Mg, Mn, and Zn reduce oxidative injury and inflammatory signaling (25).

#### 3.3.2 Astrocytes

Astrocytes are essential regulators of synaptic homeostasis and neuronal excitability in the spinal dorsal horn. ER stress in astrocytes contributes to NP by disrupting neuron-glia signaling and amplifying inflammatory responses. Mechanistically, ER stress signaling in astrocytes can initiate inflammatory cascades that enhance neuronal excitability. For instance, activation of PERK and IRE1 pathways in astrocytes induces ATF4-dependent upregulation of lipocalin-2 (LCN2) and NLRP3, which drives astrocyte-mediated inflammatory signaling and contributes to morphine tolerance and chronic pain (27). In addition, upstream regulators such as PTP1B can amplify astrocytic ER stress signaling and activate NF-*κ*B-dependent neuroinflammatory pathways in the spinal dorsal horn following nerve injury (28). Conversely, suppression of astrocytic ER stress has been shown to attenuate inflammatory signaling. The neuropeptide Phoenixin-14, for example, inhibits astrocyte inflammatory responses by targeting the eIF2*α*-ATF4-CHOP-GADD34 pathway, thereby reducing ROS production and HMGB1-mediated NLRP3 inflammasome activation (29).

#### 3.3.3 Oligodendrocytes

Oligodendrocytes maintain axonal conduction by forming and preserving myelin in the central nervous system. Disturbance of ER homeostasis in oligodendrocytes can affect myelin stability and contribute to demyelination. Studies in Wolfram syndrome, caused by mutations in the ER-related gene WFS1, show white matter loss and abnormal optic nerve conduction, suggesting that ER dysfunction may influence oligodendrocyte function and myelin integrity (30). Evidence from multiple sclerosis models further indicates that inflammatory signals such as IFN-*γ* activate ER stress responses in oligodendrocytes through the PERK-eIF2*α* pathway, which regulates oligodendrocyte survival and myelin stability (31). Considering that NP is also associated with demyelination-related pathological changes, ER stress-mediated oligodendrocyte dysfunction and demyelination may represent a potential contributing factor in the development of neuropathic pain.

### 3.4 Peripheral neurons

Peripheral sensory neurons, particularly those in the DRG and trigeminal ganglia, are essential for the development and transmission of neuropathic pain. In multiple sclerosis-associated neuropathic pain, ER stress in DRG sensory neurons, particularly through PERK signaling, disrupts intracellular Ca^2^⁺ homeostasis and alters big potassium (BK) channel activity, leading to neuronal hyperexcitability and pain hypersensitivity. Pharmacological inhibition of ER stress with 4-PBA or the PERK inhibitor AMG44 alleviates these changes and reduces NP behaviors (32). Similarly, in trigeminal NP models, ER stress promotes RUNX2-dependent upregulation of ITPR1, leading to enhanced ER calcium release, ERK activation, and increased inflammatory signaling (33). Overall, ER stress in peripheral sensory neurons disturbs calcium balance and strengthens inflammatory responses, thereby contributing to neuropathic pain.

### 3.5 Central neurons

#### 3.5.1 Spinal neurons

Spinal dorsal horn neurons are critical for the processing of neuropathic pain. Peripheral nerve injury activates ER stress and the UPR in these neurons. The ATF6 and IRE1-XBP1 pathways are particularly involved and can impair neuronal function while promoting pain hypersensitivity (34). ER stress also interacts with oxidative stress and suppresses GABAergic interneuron activity, leading to disinhibition of spinal pain circuits and central sensitization (35). In addition, nerve injury enhances store-operated calcium entry (SOCE) through STIM1, increasing intracellular calcium and strengthening glutamatergic transmission. This process further activates ER stress signaling and elevates CHOP expression (36). Pharmacological inhibition of SOCE reduces ER stress and restores synaptic balance. A similar pattern is observed in diabetic neuropathic pain, where compounds such as tanshinone IIA reduce pain by suppressing PERK-, IRE1-, and ATF6-mediated ER stress pathways (37).

#### 3.5.2 Supraspinal neurons

Supraspinal brain regions also contribute to NP processing. After peripheral nerve injury, nociceptive signals reach higher centers such as the anterior cingulate cortex (ACC), insula, prefrontal cortex (PFC), and thalamus (38). Neurons in these regions undergo functional remodeling, which is characterized by increased excitatory transmission, reduced inhibition, and enhanced network synchrony. Recent studies show that ER stress signaling contributes to these neuronal alterations. In the ACC, activation of the BiP-IRE1*α* pathway triggers downstream MAPK signaling, thereby enhancing neuronal sensitization and pain behaviors (39). ER stress also affects cognitive functions associated with chronic pain. In chronic nerve injury models, the PERK-eIF2*α*-ATF4-CHOP pathway is activated in hippocampal CA1 neurons, leading to impaired synaptic plasticity and reduced long-term potentiation (40). Inhibition of ER stress with 4-PBA restores synaptic function and improves pain-related cognitive deficits.

## 4 Neuropathic pain treatment strategies targeting endoplasmic reticulum stress

### 4.1 Chemical chaperones

#### 4.1.1 4-Phenylbutyric acid

4-Phenylbutyric acid (4-PBA) is a small-molecule chemical chaperone that stabilizes protein folding and crosses the blood-brain barrier. It reduces NP by targeting ER stress pathways (6, 54). In vasculitic peripheral neuropathy, ER stress and NF-*κ*B-mediated neuroinflammation are activated and contribute to pain, while 4-PBA suppresses these pathways and improves pain behaviors (54). In experimental autoimmune encephalomyelitis, ER stress in dorsal root ganglion neurons, particularly via the PERK pathway, disrupts Ca^2^⁺ homeostasis and BK channel function, leading to neuronal hyperexcitability. 4-PBA reverses these changes by inhibiting ER stress and restoring neuronal function (32).

#### 4.1.2 Tauroursodeoxycholic acid

Tauroursodeoxycholic acid (TUDCA) is a bile acid conjugate that inhibits ER stress and exerts cytoprotective effects (55, 56). In bone cancer pain models, activation of ER stress pathways, including PERK-eIF2*α*, IRE1, and ATF6, induces caspase-3-dependent neuronal apoptosis and contributes to pain hypersensitivity. TUDCA suppresses these pathways, reduces apoptosis, and alleviates NP (57). In dorsal root ganglion neurons, tunicamycin-induced ER stress activates GRP78 and CHOP signaling, increases oxidative stress, and triggers apoptosis. TUDCA reverses these changes by inhibiting ER stress and downstream apoptotic signaling (55). Overall, TUDCA protects neurons and reduces NP through regulation of ER stress pathways.

#### 4.1.3 Epoxyeicosatrienoic acids and soluble epoxide hydrolase inhibitors

Epoxyeicosatrienoic acids (EETs) and soluble epoxide hydrolase inhibitors (sEHi) have recently emerged as promising modulators of ER stress-mediated neuropathic pain. EETs are endogenous lipid mediators derived from the cytochrome P450 branch of the arachidonic acid cascade and possess potent anti-inflammatory and analgesic properties (58, 59). However, these bioactive lipids are rapidly degraded by soluble epoxide hydrolase (sEH). Thus, pharmacological inhibition of sEH stabilizes EETs and enhances their biological activity. Increasing evidence indicates that EETs act as upstream modulators of ER stress pathways, suppressing UPR activation and reducing ER stress-associated inflammatory responses.

In diabetic neuropathy models, inhibition of sEH reverses both ER stress markers and pain behaviors, while chemical induction of ER stress rapidly produces hyperalgesia that can be attenuated by sEH inhibitors or chemical chaperones (60). Similarly, in central post-stroke pain models, stabilization of EETs suppresses ER stress-induced reactivity of glial cells and interrupts the positive feedback loop between ER stress and neuroinflammation that drives central sensitization (15). The development of potent sEH inhibitors such as EC5026, which has progressed to early human clinical trials with favorable safety profiles (61), further highlights the therapeutic potential of targeting the EET and sEH.

#### 4.1.4 Salubrinal

Salubrinal is a selective inhibitor of eIF2*α* dephosphorylation that targets the PERK-eIF2*α* branch of the UPR and shows potential in treating ER stress-related neuropathic pain.Following nerve injury, salubrinal alleviates NP by inhibiting ER stress in the injured dorsal root ganglion, particularly through modulation of the PERK-eIF2*α*-CHOP pathway (23). Salubrinal also modulates neuroinflammation in the central nervous system. In sickle cell disease pain models, free heme activates TLR4 signaling in spinal microglia, which triggers oxidative stress, ER stress, and chronic hyperalgesia. Treatment with salubrinal reduces ER stress and pain behaviors (24). Overall, modulation of the PERK-eIF2*α* pathway by salubrinal reduces both peripheral sensitization and central neuroinflammation, supporting its potential as a therapeutic strategy for neuropathic pain.

### 4.2 Phytochemicals

Natural products, particularly those from traditional Chinese medicine (TCM), have attracted increasing attention for their ability to regulate ER stress with relatively low toxicity (Table 1). These compounds can alleviate pain by modulating key ER stress-related pathways, including PERK, IRE1, and ATF6. In addition, they act through several mechanisms, such as suppressing inflammasome activation, reducing pro-inflammatory cytokines, regulating neurotransmitter systems, and promoting neuronal repair. For example, ginsenosides (47), curcumin (41) and resveratrol (42), have been shown to regulate ER stress signaling, reduce pain behaviors, and inhibit glial reactivity. Likewise, Acorus tatarinowii (48) and Psyllium (50–52) decrease ER stress markers and improve pain by inhibiting UPR signaling in spinal tissue. Overall, these findings suggest that TCM exerts analgesic effects through coordinated modulation of ER stress and related pathways, highlighting its potential as a multi-target therapeutic strategy for neuropathic pain.

**Table 1:** ER stress-related mechanisms and pharmacological properties of traditional Chinese medicines in neuropathic pain.

Component	Related Conditions	Mechanisms of Action	Functional Characteristics	References
Curcumin	Diabetic Peripheral Neuropathy	1. Inhibits key ER stress (ERS) pathways, including the IRE1*α*–NF-*κ*B pathway and PERK–CHOP pathway. 2. Upregulates GRP78 to enhance ER homeostasis. 3. Reduces pain sensitization by limiting nociceptive transmission.	Anti-inflammatory, ER-stabilizing, and analgesic effects; modulates ERS–inflammation crosstalk.	(41)
Resveratrol	Chronic Constrictive Injury	1. Activates SIRT1 to regulate ER stress. 2. Inhibits PERK–eIF2α–ATF4 pathway, reducing neuronal apoptosis. 3. Suppresses glial activation and disrupts the inflammation–pain cycle.	Neuroprotective, anti-inflammatory, and analgesic; SIRT1-dependent ERS regulation.	(42–44)
Ginseng (Rg1, Rg2, Compound K)	Chronic Constrictive Injury	1. Downregulates TNF-α, IL-1β, IL-6 and inhibits NLRP3 inflammasome. 2. Promotes ATF6 activation and sXBP1 production to enhance ERAD.	Multi-pathway regulation with anti-inflammatory, neuroprotective, and analgesic actions.	(45–47)
Acorus tatarinowii (α-Asarone)	Chronic Constrictive Injury	1. Reduces ERS marker expression and suppresses UPR activation. 2. Activates LXR-dependent pathways linking lipid metabolism and ER function. 3. Relieves mechanical hyperalgesia.	Antioxidant, anti-inflammatory, neuroprotective, and analgesic; involves LXR–ERS crosstalk.	(48)
Psyllium (Aucubin)	Paclitaxel-Induced Peripheral Neuropathy	1. Protects Schwann cells by inhibiting ER stress. 2. Reduces apoptosis and preserves nerve fiber integrity. 3. Provides prophylactic analgesia and delays hyperalgesia progression.	Antioxidant, anti-inflammatory, and neuroprotective; suitable for prophylactic use.	(49–52)
Ershiwuwei Shanhu Pill	Cerebral Ischemic Injury	1. Modulates GRP78/XBP1/CHOP signaling to prevent ERS-mediated apoptosis. 2. Offers neuroprotection with potential value for central neuropathic pain.	ERS-mediated anti-apoptotic neuroprotection; potential application in central NP.	(53)

### 4.3 Pharmaceuticals

#### 4.3.1 Dexmedetomidine

Dexmedetomidine (Dex) is a selective *α*2-adrenergic agonist with broad-spectrum effects, including analgesic, sedative, and anxiolytic properties. In recent years, Dex has attracted increasing attention due to its neuroprotective effects (62). Notably, its neuroprotective actions are closely associated with modulation of ER stress signaling. Dex inhibits the IRE1*α*/NF-*κ*B/CHOP pathway, which reduces CHOP-mediated neuronal apoptosis and shifts the Bax/Bcl-2 balance toward cell survival, thereby improving neuronal function and limiting injury (63). Dex also inhibits ER stress-related JNK signaling, thereby decreasing neuroinflammation, neuronal injury, and excessive autophagy after cerebral ischemia-reperfusion injury (64). In addition, Dex alleviates NP by modulating UPR-dependent ER stress in the spinal cord. It enhances ER-phagy, which helps restore ER homeostasis and reduces neuronal sensitization. As a result, Dex improves nociceptive hypersensitivity in spinal nerve ligation models (65). Taken together, current evidence indicates that Dex provides both neuroprotective and analgesic effects through coordinated regulation of ER stress-related pathways.

#### 4.3.2 Ketamine

Ketamine, an NMDA receptor antagonist, is widely used in clinical practice for the management of refractory NP (66). Recent studies indicate that, beyond NMDA receptor blockade, ketamine also modulates ER stress pathways involved in pain processing. Ketamine inhibits the STING/TBK pathway, which reduces ER stress and promotes ER-phagy (67), thereby helping restore ER homeostasis. Ketamine also suppresses the ATF6 branch of the UPR. This inhibition decreases the expression of NR2B-containing NMDA receptors and reduces neuronal excitability (68). By regulating ER stress-related pathways, ketamine attenuates neuronal sensitization and improves nociceptive hypersensitivity, highlighting the importance of ER stress modulation in its analgesic mechanism.

### 4.4 Non-Pharmacological interventions

#### 4.4.1 Electroacupuncture

Electroacupuncture (EA) combines traditional acupuncture with electrical stimulation and is widely used for chronic pain (69). Recent evidence shows that EA also regulates ER stress, particularly in the anterior cingulate cortex. In this region, EA inhibits the BiP-IRE1*α* branch of the UPR and reduces downstream p38 and JNK signaling. As a result, neuronal sensitization is decreased and pain hypersensitivity is alleviated (39). In parallel, Fu's subcutaneous needling (FSN) represents another acupuncture-based approach that modulates ER stress signaling. FSN primarily acts in the peripheral nervous system. It suppresses UPR-related ER stress pathways in the dorsal root ganglion and sciatic nerve. This effect reduces inflammation, promotes axonal repair, and improves nociceptive hypersensitivity in chronic constriction injury models (70). Taken together, these findings indicate that acupuncture-based therapies can alleviate NP through coordinated regulation of ER stress pathways in both central and peripheral nervous systems.

#### 4.4.2 Pulsed radiofrequency

Pulsed radiofrequency (PRF) is a minimally invasive neuromodulation technique that modulates neural excitability through high-frequency electrical fields while avoiding thermal injury (71). In addition to its neuromodulatory effects, recent studies suggest that PRF can alleviate NP partly by regulating ER stress signaling in the spinal cord. Mechanistically, PRF increases the expression of mitsugumin-53 (MG53), a protein involved in cellular stress responses. Upregulation of MG53 suppresses the ATF4-CHOP branch of the UPR, which reduces ER stress-induced microglial inflammatory responses and neuroinflammation. Through this mechanism, PRF ultimately attenuates NP in chronic constriction injury models (72).

#### 4.4.3 Transcranial magnetic stimulation

Repetitive transcranial magnetic stimulation (rTMS) is a noninvasive neuromodulation technique that can relieve chronic and NP by regulating cortical excitability and neural networks (73). Recent studies suggest that rTMS may also influence ER stress-related pathways, including the expression of molecular chaperones such as GRP78, thereby providing neuroprotective effects (74). However, direct evidence linking rTMS-induced analgesia to ER stress modulation remains limited. Current findings indicate that rTMS may alleviate NP through regulation of ER stress signaling, but the underlying mechanisms still require further investigation.

## 5 Discussion

Although substantial progress has been made in understanding the role of ER stress in NP, several critical challenges remain. One major limitation lies in the lack of pathway specificity. The UPR regulates essential cellular functions, and broad inhibition of pathways such as PERK or IRE1*α* may disrupt physiological processes, including protein synthesis and metabolic regulation (75), thereby raising concerns about systemic toxicity. Another challenge is the complexity of ER stress signaling itself. The PERK, IRE1*α*, and ATF6 branches do not act independently but instead form a highly interconnected network. As a result, inhibition of one branch may trigger compensatory activation of others, which can reduce therapeutic efficacy and complicate the development of single-target interventions.

In addition, the translation of ER stress-targeted therapies is limited by delivery barriers and model-related issues. Effective delivery of ER stress modulators to the central nervous system remains difficult due to the blood-brain barrier (76). Many promising compounds demonstrate efficacy in preclinical models but fail to achieve sufficient bioavailability in clinical settings. At the same time, there is a substantial gap between preclinical findings and human NP. Most current evidence is derived from relatively uniform animal models, such as spinal nerve ligation, chronic constriction injury, diabetic neuropathy, or chemotherapy-induced neuropathy. In contrast, human NP is far more heterogeneous, with marked variation in etiology, symptom profile, disease duration, and comorbidities. Moreover, preclinical studies mainly assess evoked pain responses, such as mechanical allodynia and thermal hyperalgesia, whereas patients often suffer from spontaneous pain, sensory deficits, sleep disturbance, and emotional-cognitive dysfunction. Together, these limitations may reduce the translational relevance of ER stress-targeted strategies and highlight the need for more precise, clinically relevant approaches.

Future research should therefore focus on developing multi-target strategies that modulate ER stress in a balanced manner. Combination therapies that fine-tune different branches of the UPR may offer improved efficacy while minimizing adverse effects. Precision medicine approaches, including the identification of ER stress-related biomarkers, may help stratify patients and guide individualized treatment. Advanced drug delivery systems, such as nanoparticle-based carriers, may further enhance targeting efficiency. Understanding temporal dynamics is equally important. ER stress may play different roles during acute and chronic phases of NP. Identifying these windows may improve treatment timing and outcomes.

## 6 Conclusion

ER stress-targeted therapies show strong potential for the treatment of NP. They may provide both analgesic and neuroprotective benefits. Future approaches should move toward precise, cell-specific, and multi-target regulation. Addressing these challenges will be essential for translating ER stress-based therapies into effective treatments for NP.
